# Multi-Frequency Target Detection Techniques for DVB-T Based Passive Radar Sensors

**DOI:** 10.3390/s16101594

**Published:** 2016-09-28

**Authors:** Tatiana Martelli, Fabiola Colone, Enrico Tilli, Annarita Di Lallo

**Affiliations:** 1 DIET Department, Sapienza University of Rome, Rome 00184, Italy; fabiola.colone@uniroma1.it; 2 Leonardo Company, Via Tiburtina km 12,400, Rome 00131, Italy; enrico.tilli@leonardocompany.com (E.T.); annarita.dilallo@leonardocompany.com (A.D.L.)

**Keywords:** passive radar, multi-frequency, target detection, non-coherent integration, DVB-T signals

## Abstract

This paper investigates the possibility to improve target detection capability in a DVB-T- based passive radar sensor by jointly exploiting multiple digital television channels broadcast by the same transmitter of opportunity. Based on the remarkable results obtained by such a multi-frequency approach using other signals of opportunity (i.e., FM radio broadcast transmissions), we propose appropriate modifications to the previously devised signal processing techniques for them to be effective in the newly considered scenarios. The resulting processing schemes are extensively applied against experimental DVB-T-based passive radar data pertaining to different surveillance applications. The obtained results clearly show the effectiveness of the proposed multi-frequency approaches and demonstrate their suitability for application in the considered scenarios.

## 1. Introduction

Passive radar (PR) sensors exploit existing transmitters as illuminators of opportunity to perform target detection, localization, and possibly imaging [1,2]. A receiving system is appropriately designed to collect the echoes reflected from targets and sited to provide coverage of specific areas. Since there is no need to build and to operate dedicated transmitter equipment, PR sensors offer a number of advantages such as contained costs of realization and maintenance, possible covert operation, and low vulnerability to electronic countermeasures. Moreover, they do not generate additional electromagnetic pollution and have a limited impact on the landscape thanks to their reduced size, therefore they are usually referred to as “green systems” and can be employed in areas where conventional radars could not be deployed (e.g., in close proximity to residential areas).

Different transmitters for telecommunications, radio navigation, and remote sensing applications can be potentially used in this opportunistic manner, however broadcast transmitters are usually preferred solutions for surveillance purposes owing to their wider coverage. Nowadays the transmissions for Digital Video Broadcasting-Terrestrial (DVB-T) represent one of the most attractive choices as they offer a quite stable range resolution of about 20 m (equivalent monostatic value) thanks to the orthogonal frequency division multiplexing (OFDM) modulation based on persistent sub-carriers that span a total bandwidth of up to 8 MHz. Moreover, by employing an OFDM modulation, the DVB-T signals are noise-like waveforms, thus, they provide more attractive ambiguity function properties that are nearly independent of the signal content and almost time-invariant [3,4,5,6,7,8,9].

The potential of DVB-T-based PR sensors has been recently investigated for both air traffic control (ATC) and coastal/maritime surveillance applications. With reference to the first application, the performance of such systems suffers from the vertical-plane radiation patterns of typical DVB-T transmitters that are tailored to avoid wasting too much power above the horizontal plane. In fact DVB-T-based PR has been shown to allow a reliable detection capability, mainly against targets flying at low altitude, at distances up to tens of kilometers [8,9,10,11,12,13,14,15]. Therefore, such sensors can be fruitfully integrated, with the role of gap-fillers, within conventional ATC solutions to provide continuous coverage.

The exploitation of DVB-T-based PR in maritime surveillance applications has a number of positive implications and has recently attracted significant interest from the passive radar community [16,17,18,19,20]. For instance, compared to the ATC application, the expected radar coverage benefits from the longer integration times allowed by vessels and from the vertical pattern of the transmitting antennas. This implies the possibility of employing DVB-T-based PR sensors for monitoring typical maritime traffic as well as for detecting small boats close to the coast [16,17,18]. In contrast, the wide horizontal beam and high power level of the broadcast transmitters allows the availability of such signals even at large distances from the coast and this enables their exploitation for short range surveillance by PR receivers on moving maritime platforms [20]. Furthermore, the low carrier frequencies of the considered waveforms, especially when channels in the VHF or low UHF bands are exploited, might allow the detection of targets beyond the normal line-of-sight horizon thanks to the anomalous propagation phenomena typical of sea paths where super-refractive conditions are prevalent [19].

Obviously, target detection performance depends highly on the selected transmitter of opportunity and, in particular, it significantly varies with the employed DVB-T channel. In fact the selection of a given DVB-T channel among those emitted by a specific illuminator of opportunity implies a number of radar parameters must be inherently fixed. These parameters include the carrier frequency and, in turn, the radiating characteristics of the transmitter (i.e., the radiated power, the transmitting antenna gain and the corresponding pattern both on the horizontal and on the vertical planes, the adopted polarization, and the employed transmission mode among those specified by the DVB-T standard). As is well known, according to a conventional bistatic radar equation, these parameters directly affect the power level of the radar echo received from a point-like target. Moreover the parameters above further influence the receiver sensitivity as they concur to determine the backscattering characteristics for a complex target as well as the propagation channel electro-magnetic (EM) conditions, there including the dominant propagation mechanism and the possible presence of strong multipath and interfering transmissions. Due to the wide frequency band assigned to the DVB-T broadcast service (i.e., the upper portion of the very high frequency (VHF) band and the interval from 470 MHz to 862 MHz in the ultra high frequency, UHF, band), a large diversity could be experienced in terms of the above conditions and these might be time-varying during the radar operation. Therefore, in order to enhance the reliability of the considered PR system, proper strategies should be adopted aimed at improving the target detection performance by mitigating its instability tendencies.

When exploiting broadcast transmitters, a tempting solution is offered by the joint exploitation of multiple signals emitted by the same illuminator of opportunity at different frequency channels. This possibility was investigated in [21,22] with the aim of achieving finer range resolutions thanks to the widening of the resulting frequency bandwidth. To this purpose, coherent integration approaches were adopted together with advanced signal processing techniques to counteract the effects arising from the possible wide separation of channels in the frequency domain.

In contrast, in [23] the concept of a non-coherent multi-frequency (MF) integration was introduced for a FM radio-based PR as a means to mitigate the time-varying characteristics of the exploited waveforms, which largely depend on the radio program content. Besides the expected improvement due to non-coherent integration of target echoes received on multiple frequency channels, the proposed MF operation was shown to yield a remarkable advantage with respect to a single frequency (SF) approach when operating in a severe interference scenario. Compared to a coherent integration, this advantage was obtained without requiring specific properties of the received signals nor setting demanding constraints on the receiver design, so that the proposed approach was demonstrated to be a practical solution for robust and effective PR based on FM radio signals.

In this paper we aim at extending the range of applications of the MF operation presented in [23]. Specifically, its feasibility is investigated for a DVB-T-based PR sensor in order to increase the reliability of the resulting system. To this purpose different MF integration strategies are considered and proper modifications are proposed for these techniques to be effective in the newly considered application. A comparative analysis is first carried on by means of simulations, with reference to a set of specific case studies; the reported results give some insights into the properties of the proposed MF approaches. However, a full validation of the effectiveness and of the relative performance of the different multi-frequency techniques is provided through the application against live scenarios. To this purpose the conceived approaches are extensively validated against experimental data accounting for aerial and maritime surveillance applications. The reported experimental results are the fruit of a long-term collaboration between Leonardo S.p.A. (formerly Finmeccanica S.p.A., Italy) and the research group at Sapienza University of Rome. The experimental analysis allowed us to characterize the improvement yield by the MF operation in practical cases and to identify clearly the additional benefits and potential limitations of the MF detection schemes in the considered applications.

The paper is organized as follows: in Section 2, the main blocks of the DVB-T-based PR basic processing scheme are briefly illustrated. The MF detection schemes employed are described in Section 3, together with the required modifications that make them suitable for the DVB-T-based PR case. Section 4 reports the results of a comparative analysis of the theoretical performance obtained by simulations. The test campaigns and the corresponding experimental results obtained for the aerial surveillance application are illustrated in Section 5, together with an extensive analysis of the detection performance improvement provided by the MF schemes compared to the SF approaches. Section 6 is dedicated to an in-depth discussion of the outcomes obtained in experimental tests performed in a maritime scenario. Finally, our conclusions are drawn in Section 7.

## 2. DVB-T Based Passive Radar Signal Processing Scheme

**Figure 1:**
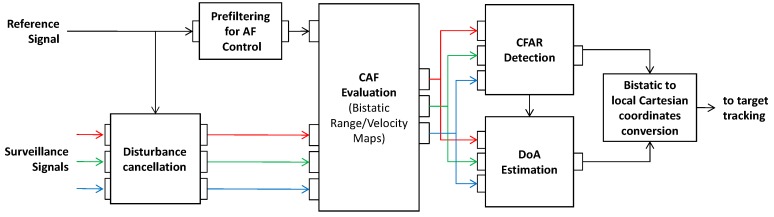
Block diagram of the DVB-T based PR processing scheme.

Figure 1 sketches the block diagram of a basic PR processing scheme to be adopted against a single DVB-T frequency channel. The main blocks relevant to target detection and localization are briefly illustrated below.

With reference to a basic PR system, the low power signal reflected from the target is collected by the main receiver (typically known as the surveillance channel) using a directive antenna steered toward the surveillance area. Multiple surveillance channels (connected to multiple antenna elements) might be implemented in order to provide spatial diversity and to gain an angular discrimination capability against the received target echoes. Since the transmitted signal is not known at the receiver, an auxiliary PR receiver (typically known as the reference channel) is usually connected to an additional directive antenna steered toward the transmitter.

The signal collected at the reference channel is first used to remove undesired contributions received together with the target echoes at the *K* available surveillance channels (i.e., direct signal from the transmitter and reflections from stationary obstacles). To this purpose, we exploit the extensive cancellation algorithm (ECA) which provides an effective cancellation filter for PR by resorting to a least square approach [24].

After the cancellation stage, the output signals $s_{k}\left(t\right)\;\left(k=1,..,K\right)$ from the ECA filter are processed with a properly mismatched [3] reference signal $s_{ref}\left(t\right)$ to evaluate the bistatic range/velocity Cross-Ambiguity Function (CAF). Assuming that the signals are sampled at frequency $f_{s}$, the resulting range/velocity maps can be expressed in discrete time notation as:

(1) $$\chi_{k}\left[l,m\right]=\overset{N_{int}-1}{\underset{n=0}{\sum }}s_{k}\left[n\right]s_{ref}^{*}\left[n-l\right]e^{-j2\pi \frac{nm}{N_{int}}}$$

where *N_int_* is the number of integrated samples and defines the coherent processing interval (CPI) $T_{int}=N_{int}/f_{s}$, *l* represents the range bin corresponding to relative bistatic range $r_{l}=cl/f_{s}$, and *m* represents the velocity bin corresponding to bistatic velocity $v_{m}=\left(\frac{mf_{s}}{N_{int}}\right)\left(\frac{c}{f_{C}}\right)$, being *c* the speed of light and $f_{C}$ the carrier frequency. The integration time ($T_{int}$) is carefully selected to limit the expected target range and Doppler migration based on the considered surveillance application [25]. For instance, in a maritime scenario, the low velocity of potential targets allows one to increase the $T_{int}$ duration up to a 1–2 s without experiencing any significant migration; this in turn allows one to obtain an acceptable SNR gain to be effectively exploited to improve the detection of small (low radar cross-section) targets or to extend the radar coverage. Increasing $T_{int}$ also allows one to improve the Doppler resolution as well as the capability to discriminate between slowly moving vessels and docked boats. In contrast, for aerial surveillance applications, $T_{int}$ on the order of fractions of seconds are used to avoid migration effects, unless some advanced approach can be exploited to compensate for such effects. Typically, the evaluation of the CAF represents the most costly operation, which might prevent the real-time operation of the PR. In this paper, the Correlation FFT technique is employed to accomplish this task since it represents the most efficient solution in the considered applications [26]. Obviously, to reduce further the computational load, sub-optimum techniques can be used to evaluate the CAF with limited SNR loss [26,27,28].

As previously mentioned, the reference signal is properly filtered before it is employed for CAF evaluation. Such a filtering stage is intended to remove the high side-lobes and spurious peaks appearing in the DVB-T signal ambiguity function (AF) [3]. To this end we resort to the linear approach presented in [9] which is based on the cascade of the pilot signals’ equalization and a Residual Peaks Removal (RPR) filter to remove the zero-Doppler peaks. Notice that, when we are interested in slowly moving target detection, the pilots equalization stage can be neglected as it allows removal of the side-peaks appearing at quite high Doppler values. However, this stage is required when aerial targets are also present in the considered scenario. Moreover, in [9] we show that pilot equalization can be obtained via a simple subtraction of a properly delayed replica from the original reference signal, which clearly represents a cost-effective solution. However, we observe that the AF sidelobe control is an essential processing step for the considered applications because a severe masking effect is expected by the strong target returns on the weak target echoes. Therefore, a wide dynamic range should be guaranteed. In this regard, it is worth noticing that, when applied against real data, the proposed AF control technique allows one to limit the sidelobe level down to 45 dB from the peak throughout the surveillance area defined on the range/velocity map.

For a SF operation, once the CAF has been evaluated at all the available surveillance channels, a conventional Cell Average Constant False Alarm Rate (CA-CFAR) threshold is separately applied to each map to detect targets with a given probability of false alarm. In this paper, a circular symmetric complex Gaussian zero mean PDF is assumed for the interference in all the considered scenarios. Recent studies [29,30] have shown that it is a quite accurate assumption for passive radar based on DVB-T signals at least with reference to the range-velocity CAF regions appearing at non-negligible Doppler values. At very low Doppler frequencies, alternative models would be better suited and, consequently, alternative detectors optimized for such models would provide enhanced results. However this is beyond the scope of this paper and could be considered as a future step for this research.

After the first detection stage, a M-out-of-N criterion can be applied to integrate the detection results obtained at the surveillance channels thus allowing a reduction in the number of false alarms. Then, assuming that the surveillance receiving channels are connected to a few horizontally aligned antenna elements, an interferometric approach can be used to estimate the direction of arrival (DoA) of the detected target echo. Finally, the bistatic range and the DoA information are converted into local Cartesian coordinates. In addition, a tracking algorithm can be applied to reduce the false tracks while yielding more accurate measurements. In order to improve the detection performance of the proposed system, in this paper we consider the joint exploitation of multiple DVB-T frequency channels according to appropriate schemes as illustrated in the following section.

## 3. Multi-Frequency Detection Schemes

We assume that each receiving antenna simultaneously collects *N* radio frequency channels, all transmitted by the same transmitter. Then we apply to each frequency channel the first two processing stages described in Section 2, thus obtaining *NK* different outputs, namely the CAF maps $\chi_{k,n}\left[l,m\right]\;\left(k=1,..,K,\;n=1,..,N\right)$ evaluated at *K* surveillance channels for *N* different frequency channels. The analysis conducted in [23] against FM radio-based PR data demonstrated that quite effective and robust MF detection schemes are provided by:

(1) Resorting to a centralized linear integration followed by a CA-CFAR detection technique, or
(2) using a binary integration after applying a first-decision threshold separately on each frequency channel output.

As is apparent, both approaches exploit a non-coherent integration across frequency channels whose potentialities are well known in the radar literature [31,32] where typically this is used to: (i) increase the detection performance by exploiting multiple target echoes received with the same SNR on multiple channels; (ii) average over the fluctuation of the target radar cross section (especially when using frequency agility). However, for the MF PR, we also aim to exploit the non-coherent integration to make detection schemes robust with respect to the undesired characteristics of the received waveforms. When FM radio broadcast transmissions are exploited such undesired characteristics are typically due to the time-varying nature of both the signal AF and of the propagation channel EM conditions (i.e., multipath effects, presence of co-/adjacent-channel interfering transmissions).

Based on the successful results obtained in the FM radio-based PR case, we selected the above MF approaches for potential application to the DVB-T-based PR. The resulting detection schemes are briefly summarized below followed by a detailed discussion of the modifications required by the exploitation of the DVB-T broadcast transmissions:

*Centralized MF detection scheme (CEN-MF-N)*: Basically such an approach operates on single surveillance antennas by incoherently adding (after square law detector) the corresponding CAFs obtained at different frequency channels:

(2) $$z_{k}\left[l,m\right]=\overset{N}{\underset{n=1}{\sum }}{\left|\chi_{k,n}\left[l,m\right]\right|}^{2}$$

and then comparing the cell under test $z_{k}\left[l_{0},m_{0}\right]$ with the average intensity estimated over *Q* secondary data surrounding the cell under test ($z_{k}\left[l_{q},m_{q}\right],\;q\in I_{\left[l_{0},m_{0}\right]},\;\left|I_{\left[l_{0},m_{0}\right]}\right|=Q)$:

(3) $$z_{k}\left[l_{0},m_{0}\right]\begin{array}{c} H_{1}\\ ≷\\ H_{0} \end{array}G_{C}\cdot \left(\underset{q\in I_{\left[l_{0},m_{0}\right]}}{\sum }z_{k}\left[l_{q},m_{q}\right]\right)$$

where, under the Gaussian assumption for the disturbance affecting each map, the threshold $G_{C}$ is readily found by inverting the following expression of the theoretical false alarm probability:

(4) $$P_{fa}=\sum_{n=0}^{N-1}\left(\begin{array}{c} QN+n-1\\ n \end{array}\right){\left(\frac{G_{C}}{QN}\right)}^{n}{\left(1+\frac{G_{C}}{QN}\right)}^{-QN-n}$$

Along with the expected enhancement of the SNR on the resulting integrated maps, with this approach the characteristics of a bad frequency channel are likely to be averaged with the good ones, thus yielding a limited impact on the final performance.

*Decentralized MF detection scheme L/N (DEC-MF-L/N*): In this case, a detection is declared at a given range/velocity location when *L* detections out of N channels are obtained for the considered pixel over the single-channel maps. Specifically, by defining the binary map at the output of the first threshold application:

(5) $$b_{k}\left[l_{0},m_{0}\right]=\left({\left|\chi_{k,n}\left[l_{0},m_{0}\right]\right|}^{2}>G_{D}\underset{q\in I_{\left[l_{0},m_{0}\right]}}{\sum }{\left|\chi_{k,n}\left[l_{q},m_{q}\right]\right|}^{2}\right)$$

the DEC-MF-L/N the detection scheme is obtained as:

(6) $$\overset{N}{\underset{n=1}{\sum }}b_{k}\left[l_{0},m_{0}\right]\begin{array}{c} H_{1}\\ ≷\\ H_{0} \end{array}L$$

In this case a conventional CA-CFAR threshold $G_{D}$ is separately applied at each frequency channel where the $P_{fa}^{SF}$ is properly set in order to achieve the desired $P_{fa}$ after the MF binary integration:

(7) $$P_{fa}=\sum_{i=L}^{N}\left(\begin{array}{c} N\\ i \end{array}\right)P_{fa}^{{SF}^{i}}{\left(1-P_{fa}^{SF}\right)}^{N-i}$$

Obviously, this approach yields reasonable detection performance improvement when at least *L* of the *N* exploited channels are characterized by a reasonable SNR for a given target. However, compared to the centralized approach, it has the potential to discard strong undesired peaks appearing in the range/velocity maps at single frequency channels. Basically, as the integration is performed after a first thresholding stage, it operates against binary outputs so that the contribution of each frequency channel, at each range/velocity pixel, is quantized to 0/1 values, regardless of the original amplitude measured at that pixel. This could represent an interesting characteristic against intentional/unintentional interference yielding ghost targets, as discussed later in this paper.

### 3.1. Modifications Required for Application Against DVB-T Signals

Regardless of the detection scheme employed, for the MF integration to be effective, the target echoes received at different frequency channels are supposed to be perfectly aligned on the range/velocity plane. This is typically the case if: (i) the same bistatic geometry is exploited, namely all the frequency channels are broadcasted by the same transmitter and received by the same PR receiver; (ii) the target size is limited with respect to the radar range resolution; and (iii) anomalous propagation effects do not take place or, if present, they yield quite similar propagation paths on the integrated frequency channels.

In addition, from a discrete signal processing point of view, the range/velocity CAF maps have to be evaluated on common grids to allow a pixel-by-pixel integration of the results obtained at different frequency channels (either the intensity levels or the binary outputs after the application of the first-decision threshold).

The above issues could be easily managed in a FM radio-based PR [23]. In contrast, the finer range resolution and the wider frequency separation of DVB-T channels might jeopardize the validity of the above assumptions. Therefore, these assumptions have to be carefully verified and proper strategies should be devised to make the system robust to potential limitations. A dedicated discussion of these problems is reported in the following sub-sections distinguishing between limitations arising at a signal processing stage and limitations due to the geometrical and electromagnetic characteristics of the acquisition scenario.

#### 3.1.1. CAF Maps Evaluation on Common Bistatic Range/Velocity Grids

Aiming at effectively integrating the target echoes received at the available DVB-T channels, the range-velocity maps obtained at different frequency channels should be directly comparable, i.e., they should be evaluated on common grids both in range and in velocity.

As regards the range dimension, the same pixel spacing is guaranteed by the common sampling frequency provided that the same clock is exploited or that the clocks regulating different receiving channels are properly synchronized.

The pixel spacing in the velocity dimension is a function of the CPI length $T_{int}$ and of the carrier frequency. In [23] the use of different integration times was suggested at the N frequency channels in order to guarantee a constant spacing across different channels, according to the following criteria:

(8) $$\Delta v=cost\to T_{int_{n}}=\frac{c}{\Delta vf_{C_{n}}}=\frac{\lambda_{n}}{\Delta v}\;\text{with}\;n=1,\ldots ,N$$

where $\lambda_{n}=c/f_{C_{n}}$ is the wavelength of the *n*-th carrier.

The approach above can be shown to be robust and effective in the FM radio based PR case thanks to the narrowness of the frequency band assigned to the analog radio broadcast (i.e., the interval 88–108 MHz) and to the possibility to freely select the integration time. In fact, by applying Equation (8), slightly different integration times are obtained even operating with the extreme channels of the allowed frequency band. Specifically, the largest ratio $T_{int_{n}}/T_{int_{m}}$ across the band is approximately 1.23, which yields a resulting difference in term of SNR gain smaller than 0.9 dB and a possible increase in term of computational load smaller than 25% for typical CPI lengths [26].

In contrast, when DVB-T signals are exploited, the setting in Equation (8) does not necessarily represent a correct approach. This is due both to the wider frequency band assigned to DVB-T transmissions (spanning from 470 MHz to 862 MHz) and to the constraints regulating the selection of the CPI length.

With particular reference to the latter point, we recall that the DVB-T-based PR processing scheme in Figure 1 include, as an essential step, the application of proper techniques aimed at the control of the DVB-T signal AF (i.e., for the removal of typical high side-lobes and spurious peaks appearing in the range/velocity map and limiting the weak target detection). However, for a correct operation of the adopted techniques, the integration time must be selected as an integer multiple of a super-symbol (i.e., a period of 4 OFDM symbols) [9]. This implies that, following the approach in Equation (8) and later adjusting the selected value, the actual integration time $T_{int}^{\prime }$ at the *n*-th channel would be:

(9) $$T_{int_{n}}^{\prime }=\left\lfloor T_{int_{n}}/T_{{ss}_{n}}\right\rceil \cdot T_{{ss}_{n}}=T_{int_{n}}+\delta T_{n}$$

where $T_{int_{n}}$ is the desired CPI length, ⌊x⌉ is the rounded integer value of *x*, $T_{{ss}_{n}}$ is the super-symbol duration with the adopted modulation parameters, and $\delta T_{n}$ is the rounding error at the *n*-th channel and it results $\left|\delta T_{n}\right|\leq T_{{ss}_{n}}/2$.

The use of $T_{int_{n}}^{\prime }$ in lieu of $T_{int_{n}}$ yields a difference $\varepsilon_{v}$ between the desired and the actual velocity pixel spacing (Δv and $\Delta v_{n}^{\prime }$, respectively):

(10) $$\varepsilon_{v_{n}}=\Delta v_{n}^{\prime }-\Delta v=\lambda_{n}\left(\frac{1}{T_{int_{n}}^{\prime }}-\frac{1}{T_{int_{n}}}\right)=\lambda_{n}\left(\frac{1}{T_{int_{n}}+\delta T_{n}}-\frac{1}{T_{int_{n}}}\right)=\Delta v\left(\frac{-\delta T_{n}}{T_{int_{n}}+\delta T_{n}}\right)$$

For a given frequency channel, the largest misalignment between the nominal and the actual grid in the velocity dimension is experienced at the highest velocity value $v_{max}$ included in the CAF map. Specifically, the maximum relative displacement with respect to the nominal pixel spacing is given by:

(11) $$E_{{rel}_{n}}=\frac{1}{\Delta v}\left\lfloor \frac{v_{max}}{\Delta v}\right\rfloor \left|\varepsilon_{v_{n}}\right|=\left\lfloor \frac{v_{max}}{\Delta v}\right\rfloor \frac{\left|\delta T_{n}\right|}{T_{int_{n}}+\delta T_{n}}$$

and assuming that $v_{max}$ is an integer multiple of Δv and that $\delta T_{n}\ll T_{int_{n}}$, we obtain:

(12) $$E_{{rel}_{n}}≅\frac{v_{max}}{\lambda_{n}}\left|\delta T_{n}\right|$$

**Figure 2:**
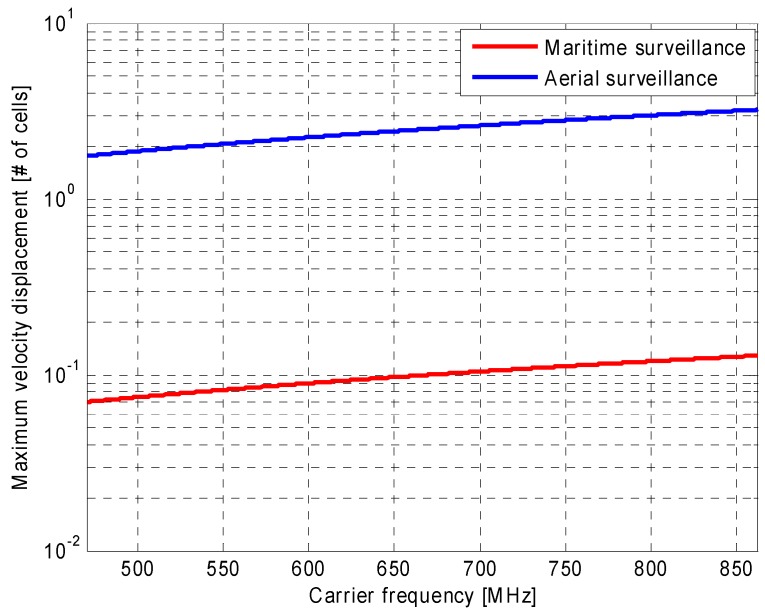
Maximum relative displacement of the CAF grid in the velocity dimension due to the rounding errors affecting the CPI length selection.

As is apparent, the maximum relative displacement $E_{{rel}_{n}}$ at the *n*-th frequency channel is a function of the carrier frequency and varies with the rounding error affecting the selection of the CPI length and with the CAF extent in the velocity dimension, which in turn depends on the considered surveillance application. The result is shown in Figure 2 assuming, as a worst case, $\left|\delta T_{n}\right|=\frac{T_{{ss}_{n}}}{2}=2.24\cdot 10^{-3}$ s for a 8 k transmission mode, a useful symbol duration $T_{U}=896\;\mu s$ and a guard interval of duration $T_{GI}=T_{U}/4$. The maximum relative displacement in Equation (12) is reported as a function of the carrier frequency for two different surveillance applications. Specifically, for the case of a maritime scenario, the maximum expected bistatic velocity for targets of interest is arbitrarily set to 20 m/s; with such position, the maximum relative displacement of the CAF grid in the velocity dimension is negligible across the UHF band. In contrast, taking $v_{max}=500\;m/s$ as for aerial surveillance applications, such a displacement might easily exceed the velocity cell thus making the proposed integration scheme completely ineffective.

In addition, it is to be noticed that, due to the large diversity among the available carrier frequencies within the DVB-T band, following the approach in Equation (8) would yield non-negligible differences among the jointly exploited frequency channels both in term of coherent integration gain and in term of computational burden. In fact, in the DVB-T case, even limiting the analysis to the UHF band, the largest ratio $T_{int_{n}}/T_{int_{m}}$ across the band would yield a difference in term of SNR gain approximately equal to 2.6 dB and a possible increase in term of computational load up to 80%. These differences cannot be neglected as they would result in a significant imbalance in the contributions provided by different integrated channels as well as in the computational resources to be assigned to each channel.

Taking into account the considerations above, in order to benefit from a MF integration scheme also in a DVB-T-based PR, we propose to modify the CPI length selection strategy with respect to the solution in [23]. Specifically, the nominal $T_{int}$ is kept constant across different frequency channels in order to guarantee the same coherent integration gain. The value of $T_{int}$ is selected so as to guarantee the desired velocity resolution at the highest carrier frequency included in the integration scheme, i.e., $T_{int}=\frac{\lambda_{min}}{\Delta v}$. This would obviously yield under-sampled grids at lower carrier frequencies.

Slight differences in the selection of the CPI length are allowed due to the rounding errors in Equation (9). Basically these errors still apply due to the constraints that regulate the selection of the CPI length; in this regard, we observe that different frequency channels might be transmitted using different transmission modes and/or modulation parameters. Consequently we observe that the rounding errors might differ from one channel to another channel even assuming the same desired CPI length. Therefore, with the proposed approach, the actual integration times $T_{{int}_{n}}^{\prime }$ at the different channels are given by:

(13) $$\begin{array}{cc} T_{int_{n}}^{\prime }=\left\lfloor T_{int}/T_{{ss}_{n}}\right\rceil \cdot T_{{ss}_{n}}=\frac{\lambda_{min}}{\Delta v}+\delta T_{n} & n=1,..,N \end{array}$$

However, as for typical values $\left|\delta T_{n}\right|\ll T_{int}$, the resulting differences in term of SNR gain are negligible. The same CAF grid in the velocity dimension is then obtained by properly oversampling the final map. This can be easily achieved by exploiting a Chirp Zeta Transform (CZT) in lieu of the standard Fast Fourier Transform (FFT). Alternatively, a simple zero-padding operation can be performed prior to applying conventional algorithms for the CAF evaluation. In fact, following the same approach of Equations (9)–(12), it can be easily verified that the quantization yield by the sampling frequency $f_{s}$ is negligible in term of maximum relative displacement of the final velocity grid. In particular, the number of zeros to be padded has to be set in order to achieve the desired velocity spacing Δv at all the available frequency channels regardless of the rounding errors:

(14) $$\begin{array}{cc} Z_{P_{n}}=\left\lfloor f_{s}\left(\frac{\lambda_{n}}{\Delta v}-T_{int_{n}}^{\prime }\right)\right\rceil =\left\lfloor f_{s}\left(\frac{\lambda_{n}-\lambda_{min}}{\Delta v}-\delta T_{n}\right)\right\rceil & n=1,..,N \end{array}$$

Therefore the same velocity spacing is obtained at all the considered frequency channels and the resulting CAF maps can be directly compared. However, the maps obtained at lower carrier frequencies turn out to be oversampled in the velocity dimension so that the target echo might span consecutive velocity bins and a white noise disturbance might show some degree of correlation across velocity bins. However, these effects are not expected to severely affect the possibility to control the false alarm rate provided that a reasonable number of secondary data is adopted by the CFAR detection scheme together with a proper guard interval. Moreover we observe that the oversampling factor is always smaller than 1.84 and reaches its maximum only when both the extreme carrier frequencies (at the right and at the left of the DVB-T band) are included in the MF integration.

#### 3.1.2. Typical Causes of Target Echo Misalignment across Channels and Counteracting Strategies

For an effective MF operation, the target echoes received at different frequency channels should be perfectly aligned on the range/velocity plane. In such condition, the proposed non-coherent integration strategies are expected to enhance the resulting SNR (centralized strategy) or, equivalently, to exploit the coincidence of detections (decentralized strategy) thus improving the target detection capability of the system.

The hypothesis above typically holds in FM radio-based PR exploiting multiple frequency channels emitted by the same illuminator of opportunity [23]. Basically, in that case, a moderate diversity is experienced in term of carrier frequencies due to the limited spectral extent of the FM radio broadcast band. Moreover, even in the presence of slight deviations from the ideal assumption, the robustness to these effects is still guaranteed by the coarse range resolution (order of km).

In contrast, the finer range resolution and the wider frequency separation of DVB-T channels determine an increased sensitivity to non-ideal conditions that might cause a range/velocity displacement of target echoes received at different frequency channels. Therefore the validity of the above assumption has to be carefully verified and proper strategies should be devised to make the system robust to potential limitations.

Among the possible causes of target echoes misalignment across frequency channels, the exact position of the transmitting antennas plays a key-role. Basically a transmitting site broadcasting multiple DVB-T channels might be composed of a few masts with multiple transmitting antennas possibly operated and maintained by different service providers. The distance between these masts can be in the order of tens of meters or more. According to the information available online, all the broadcasted DVB-T channels are labeled with the same transmitter name even if they could be emitted by largely displaced antennas. In other words, by exploiting multiple frequency channels, a pure bistatic geometry is not guaranteed so that a given target can be observed at different bistatic ranges and, possibly, at different bistatic velocity. For typical distances between transmitting antennas, the impact on the bistatic velocity is expected to be negligible compared to the velocity resolution cell usually synthesized for detection purposes. In contrast the range displacement depends on the positions of both the target and the receiver and might range from zero to a maximum value that is exactly equal to the distance between the emitting antennas (e.g., when the transmitting antennas and the target lie on a straight-line and the receiver is equidistant from both transmitters). As is apparent, this effect can be neglected in FM-based PR since the resulting displacement would be much smaller than the radar range resolution. In contrast, it would jeopardize the effectiveness of the proposed MF approach in the DVB-T-based PR case since the same displacement would cause the target echoes to occupy different range cells.

Another possible cause of range misalignment between target responses at different channels is related to the target size. In fact, if the target physical size is comparable or larger than the range resolution cell, its echo in principle might span multiple range bins. However, depending on the carrier frequency of the exploited DVB-T channel, different target portions might have different scattering characteristics. In extreme cases, the dominant target echo might be generated by distinct and largely displaced parts of the target thus yielding a range displacement of the resulting responses. This effect is obviously more evident when largely diverse carrier frequencies are employed.

Finally, the presence of anomalous propagation effects has to be mentioned as possible cause of target echo misalignment of the CAF map. For example, when super-refractive conditions or even ducting occur, the propagation paths of target echoes might differ from frequency channel to frequency channel thus yielding a significant displacement on the range/velocity plane.

All the above conditions might severely limit the effectiveness of the proposed MF approaches. Obviously, when the expected displacement is very large, computationally expensive signal processing techniques have to be applied to co-register the resulting maps prior to integrating their results. However, when the target echoes misalignment is limited to a few cells, a simple and effective approach can be devised based on the decentralized integration strategy.

Specifically, we propose to extend the integration of the binary results after the application of the first threshold so that a few consecutive range bins are included in the sum. Basically, by defining a proper window in the range dimension composed of W cells, Equation (6) becomes:

(15) $$\overset{N}{\underset{n=1}{\sum }}\left(\overset{W/2}{\underset{p=-W/2}{\sum }}b_{k}\left[l_{0}+p,m_{0}\right]>0\right)\begin{array}{c} H_{1}\\ ≷\\ H_{0} \end{array}L$$

In other words, the binary maps to be integrated are obtained by employing an “OR” condition on the detection results obtained on *W* consecutive range cells. By properly setting the window size W, limited range misalignments could be managed.

Obviously, this is paid in term of target detection performance against targets that do not yield any displacement of the corresponding echoes. In fact, with this approach, the detection threshold to be adopted at the first stage has to be properly increased in order to maintain the same final rate of false alarms. In fact, after the integration on consecutive range cells, the false alarm rate increases as:

(16) $$P_{fa}^{{SF}^{\left(W\right)}}=1-{\left(1-P_{fa}^{SF}\right)}^{W}≅WP_{fa}^{SF}$$

Moreover, the proposed solution inherently implies a range resolution degradation by a factor of W; therefore the window size has to be limited as far as possible and the application of the proposed approach has to be restricted to cases where it represents an essential step.

## 4. Theoretical Detection Performance of Multi-Frequency Approaches

A preliminary analysis of the proposed integration strategies is reported in this section in terms of theoretical detection performance. Specifically the benefits of the considered approaches are evaluated with reference to different simulated scenarios.

**Figure 3:**
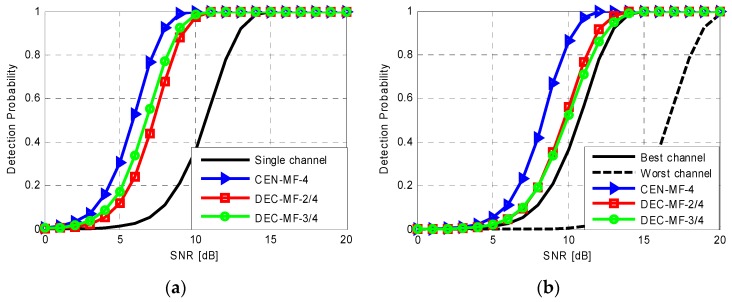
Detection probability vs. SNR obtained by means of Monte Carlo simulations for different processing approaches for *N* = 4, nominal *P_fa_* = 10^−5^ and Q = 100 secondary data for the case of: (**a**) same SNR at the N channels; (**b**) SNR decreasing of 2 dB at each channel (results as a function of the highest SNR).

Firstly, we consider the case of the joint exploitation of *N* = 4 DVB-T channels for the detection of a target showing the same SNR over the four channels. Figure 3a shows the detection probability as a function of the SNR obtained by means of Monte Carlo simulation for the proposed integration approaches operating with a CA-CFAR detection scheme (with Q = 100 and *P_fa_* = 10^−5^). Specifically the performance obtained when using a single channel is compared to the results of the centralized integration strategy and the decentralized strategy operating with *L* = 2 and *L* = 3. As is well known, a significant performance improvement is achieved with all the integration strategies due to the integrated SNR increase. The centralized linear integration yields the best performance while both the decentralized strategies with *L* = 2 and *L* = 3 suffer only of a slight loss due to the limited increase in the resulting equivalent SNR.

In practical situations, the same target can be observed with different SNR over different DVB-T channels due to: (i) different transmitted power levels; (ii) different EM behaviour of the target at different carrier frequencies; (iii) different noise floor levels due to the interference sharing the frequency band. As an example, Figure 3b reports the results obtained for a target with SNR regularly decreasing of 2 dB across the four DVB-T channels. The detection probability curves are evaluated as a function of the highest SNR and the results obtained for the best and the worst channels are reported for comparison. Only the centralized approach yields a remarkable improvement in the detection capability with respect to the best performing channel. However, it is to be noticed that also the decentralized MF schemes are able to recover the significant performance degradation provided by the worst performing channel. However, notice that, when a ghost target is present on the map of a given channel, due for example to an undesired side-peak of the employed waveform, the decentralized approach potentially yields the benefit of controlling the false target detection probability. Therefore the previously analysed drawback of this approach in terms of detection capability can result in a significant advantage in terms of ghost targets rejection.

**Figure 4:**
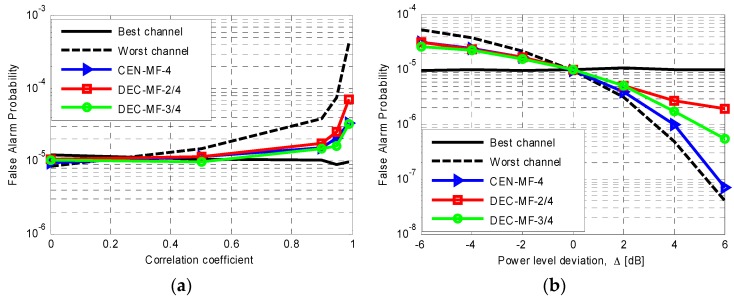
Actual false alarm probability obtained by means of Monte Carlo simulations for different processing approaches for *N* = 4, nominal *P_fa_* = 10^−5^ and Q = 100 secondary data in the case of: (**a**) exponentially correlated interference affecting the secondary data with varying one-lag correlation coefficient; (**b**) a non-homogenous region contaminating 20 secondary data in two out of the four DVB-T channels and characterized by additive noise with circular symmetric complex Gaussian zero mean PDF and a power level that deviates from the homogeneous background by Δ dB.

In the case studies considered above, a perfect control of the false alarm probability is guaranteed as the simulated disturbance was assumed to follow a circular symmetric complex Gaussian zero mean PDF with homogeneous characteristics across secondary data. However, in practical cases, the interference affecting the range-Doppler maps might deviate from this assumption and this could severely prevent an effective control of the actual false alarm rate. Specific examples along this line are shown in Figure 4 where the results of Monte Carlo simulations are reported in term of actual probability of false alarm for specific case studies.

Particularly, in Figure 4a the interference affecting the DVB-T channels is assumed to be correlated on adjacent range-velocity bins. An exponential model is used to model such correlation in the secondary data used by the CFAR detection scheme and the results are reported as a function of the one-lag correlation coefficient. With all the considered detection schemes, the actual false alarm rate coincides with the nominal value only for uncorrelated disturbance contributions (notice that this is always the case for the best single channel). In contrast, at the worst DVB-T channel, the actual false alarm rate rapidly increases, as the correlation coefficient exceeds 0.5 as the resulting secondary data correlation yields an underestimation of the detection threshold. However, the results of this simulation show that the MF approaches allow a partial mitigation of this effect since significant deviations from the nominal false alarm rate are observed only for high values of the correlation coefficient. In this case the centralized and the decentralized scheme with *L* = 3 yield comparable results.

In Figure 4b we consider the case of a non-homogenous region appearing in the secondary data in two out of the four DVB-T channels. Specifically, we assume that this region contains additive complex Gaussian noise with a power level that deviates from the homogeneous background level by Δ dB. This region contaminates 10 out of the 30 available secondary data and appears at different pixels at the two affected channels. As is apparent the false alarm rate control is guaranteed only when Δ = 0 dB (i.e., for homogeneous secondary data) when DVB-T channel are employed that are affected by such non-homogeneity. In fact, the actual false alarm probability rapidly increases/decreases as Δ deviates from 0 dB. However, again, the MF schemes allow to mitigate the effect of the non-homogeneity by providing a more stable false alarm rate. In particular, in this case, the decentralized approach shows slightly better performance with respect to the centralized MF scheme.

The comparative analysis above gave some insights into the properties of the proposed MF approaches for specific case studies. However, for a full validation of the effectiveness and of the relative performance of the different MF techniques, the application against experimental data sets is strictly required. Basically, many phenomena can occur with live data that cannot be easily simulated or even modeled but that are typical of the considered applications.

## 5. Experimental Results for Aerial Surveillance Application

In this section, we compare the detection performance provided by a SF operation and the proposed MF integration strategies against experimental data collected by a DVB-T-based PR in an aerial surveillance scenario.

### 5.1. Test Campaign and Data Collection

We report the results obtained against the data collected during the test campaigns that were carried out in November 2015 at the Leonardo S.p.A. premises, in Rome.

**Figure 5:**
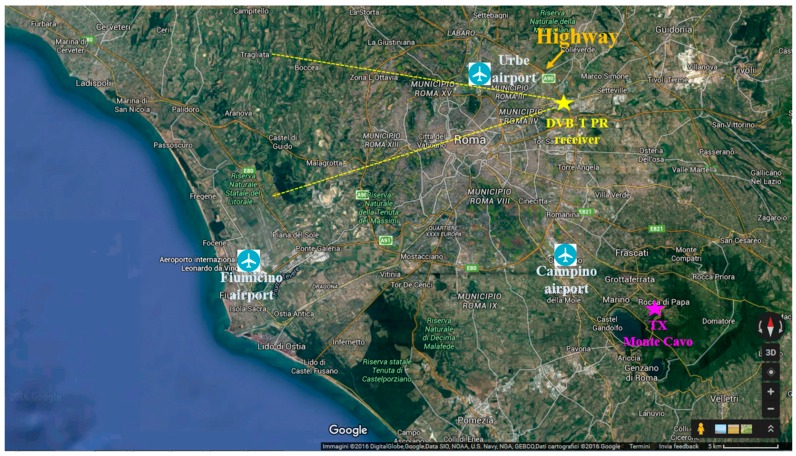
Sketch of the acquisition geometry in the case of aerial surveillance application.

The acquisition geometry is depicted in Figure 5. The reference antenna was steered toward the DVB-T transmitter of Monte Cavo (about 22.5 km away) while two surveillance antennas were adopted, pointed at 270° clockwise from north. Specifically, Yagi-Uda antennas were employed yielding a main beam width of about 36°. Therefore the main beam of the surveillance antennas included a section of the civilian air traffic departing or arriving to the Fiumicino and Ciampino airports. The corresponding aircrafts are used in the following as targets of opportunity to test the proposed MF detection techniques based on the available ATC registrations.

During the test campaign, seven different data sets have been collected. Each data set is composed by sequential data files of temporal duration equal to 1.2 s, spaced by about 4 or 5 s. The total number of data files and the total time duration of each data set is reported in Table 1. In the considered test campaign, the signals for 4 DVB-T channels within the UHF band have been simultaneously collected, all emitted by the same transmitter at the following carrier frequencies: 586 MHz, 714 MHz, 762 MHz, and 634 MHz. Specifically, among all the available digital television channels, we have selected those characterized by high power levels (79 KW, 100 KW, 35 KW and 79 KW, respectively) and that, based on a rough analysis, had the best performance for PR purposes.

First of all, in order to evaluate the performance of each DVB-T channel, all the available data files of each carrier frequency have been separately processed according to a basic DVB-T PR processing scheme described in Section 2. In particular, the ECA [24] is applied with 1000 taps (i.e., 33 km @ 𝑓𝑠 = 64/7 MHz). A CPI of 0.3 s is used to evaluate the CAF maps that span a surveillance area of 100 km × 300 m/s. Target detection is performed by resorting to a CA-CFAR threshold with a probability of false alarm equal to $P_{fa}=10^{-3}$. In detail, a region of $N_{R}=19$ cells in range and $N_{V}=5$ cells in velocity around the cell under test has been employed to estimate the disturbance, resulting in Q = 86 secondary cells also taking into account a guard interval of $N_{r}=N_{v}=3$ cells in range and velocity, respectively. Finally, a 2 out of 2 criterion is adopted to integrate the detection results obtained at the two surveillance channels allowing a nominal $P_{fa}=10^{-6}$ on the final range-velocity plane.

**Figure 6:**
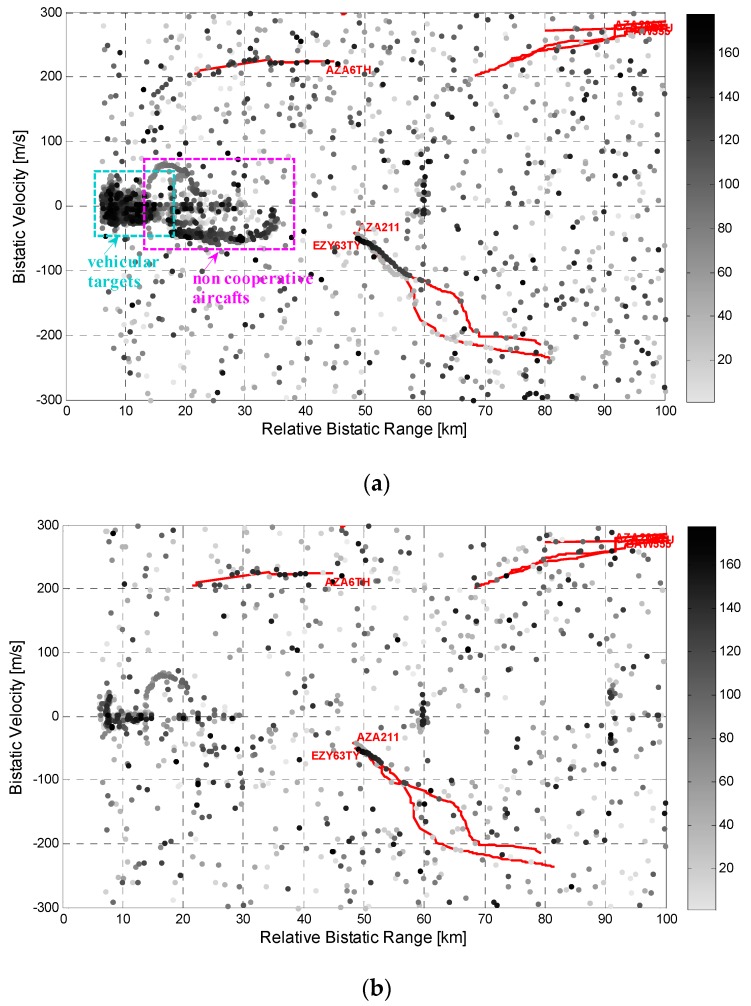
Detection results over the range-velocity plane for Data Set #1 using single DVB-T channels representing the best and the worst cases, respectively: (**a**) channel at 714 MHz; (**b**) channel at 634 MHz.

As an example, Figure 6a,b show the detection results obtained for the first data set (Dataset #1) with the DVB-T channels transmitted at 714 MHz and 634 MHz, respectively. Notice that the reported frequency channels represent the best and the worst channels in terms of detection capability based on a SF operation. In detail, all the detection results within the total acquisition time (12 min) have been reported. In particular, in each figure, the grey dots represent the raw detections of the PR sensor while in red is reported the available air truth for direct comparison. Specifically, a gray scale has been employed to plot the raw detections obtained on successive scans (i.e., the gray tone used progressively gets darker for more recent plots). For the sake of clearness, all the plots within a bistatic range of 6 km have been discarded assuming that they are due to disturbance residuals.

As is apparent, even operating with the best performing channel (see Figure 6a), the system is able to continuously detect the targets appearing between 50 and 60 km at bistatic velocities around −60 m/s, which correspond to aircraft landing at the Fiumicino airport. In contrast, just a few isolated plots are obtained for other target track portions provided by the available air-truth. This might be caused by the higher velocity of these targets that yields migration effects which might limit the coherent integration gain. Moreover, as these targets usually correspond to higher altitude targets, their detection might be prevented by the vertical-plane radiation patterns of DVB-T transmitters that are tailored to avoid wasting too much power above the horizontal.

Many detections appear within the first 16 km (highlighted by the cyan rectangle in Figure 6a) which correspond to the returns from vehicles moving on the stretch of highway near the receiver position (see Figure 5). In fact, the achieved range-velocity values are compatible with the expected range-velocity trajectory of ground moving targets travelling on the highway as shown in [14]. Moreover, other tracks appear between 14 and 35 km (highlighted by the magenta rectangle in Figure 6a). These likely correspond to aircraft, not equipped with a cooperative transponder, that are landing or taking off from the small civilian Urbe airport situated near the Leonardo premises as illustrated in Figure 5.

The comparison between the Figure 6a,b clearly shows that the target detection capability highly varies with the employed DVB-T channel due to different target backscattering properties as well as to the propagation channels EM conditions. A remarkable advantage with respect to the SF operation can be obtained by exploiting the proposed MF approaches as demonstrated in the next sub-section.

### 5.2. Extensive Analysis of the Target Detection Performance

**Figure 7:**
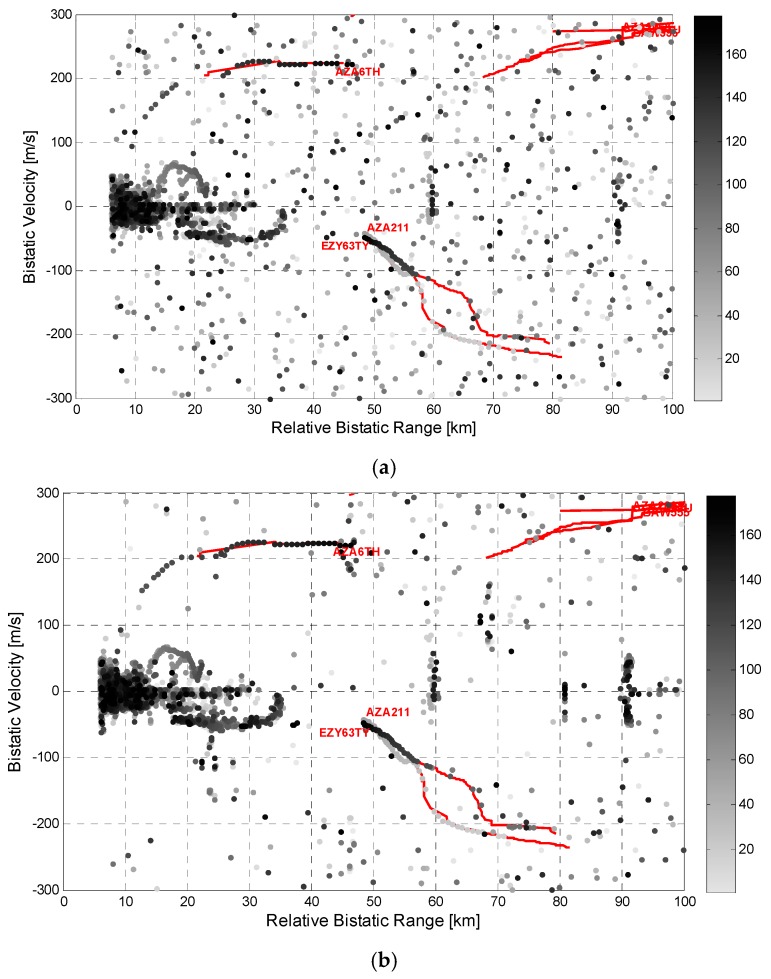
Detection results over the range-velocity plane for Data Set #1 using two different MF detection schemes: (**a**) DEC-MF-2/4; (**b**) CEN-MF-4.

As an example of the results obtained with the proposed MF integration schemes, Figure 7 reports the output of the application of these schemes against the same dataset considered in Figure 6. Specifically Figure 7a reports the detection results obtained with a decentralized detection scheme based on a 2-out-of-4 strategy (DEC-MF-2/4) which was verified to be a quite robust and effective solution among the possible decentralized strategies. Figure 7b refers to the case of a centralized integration scheme using all the available frequency channels (CEN-MF-4).

By comparing Figure 6 and Figure 7, the detection performance improvement provided by the MF approaches is quite apparent as many additional plots are obtained for the targets of opportunity.

In the following, we define a given detection as “correct” when it appears at the expected range-velocity location at the expected time instant. With this counting procedure, reasonably accurate estimates of the detection rate can be obtained because, when the false alarm rate is kept sufficiently low, it is very unlikely to include false plots within the correct detections. This procedure can be easily implemented based on the availability of accurate enough truth data (temporal interpolation can be performed against time discontinuous data for the air traffic of opportunity). All the remaining plots are then labeled as false alarms.

Based on the definitions above the improvement is found to be substantial for the long track appearing at about 220 m/s. In fact, with both the considered MF schemes, the corresponding aircraft is almost continuously detected all along the performed acquisition. Specifically, for that target, the number of correct detections moves from 12 with the worst performing single channel, to 20 using the best performing channel, to 29 with the DEC-MF-2/4, up to 35 when the CEN-MF-4. We recall that the number of possible detections for each target is equal to the available data files within the observed area (in range, velocity and angular sector), namely 44 for the considered target.

Moreover the number of false alarms appears to be significantly reduced when jointly exploiting all the available frequency channels according to one of the considered MF strategies. In this regard we recall that, when applying the proposed MF detection schemes, the detection threshold are properly set in order to achieve, after integration, the same nominal false alarm rate provided by the SF operation.

By extending the above analysis to all the available data sets, we evaluated the number of correct detections performed against all the targets of opportunity included in the available air-truth. Moreover, the actual false alarm rate has been estimated by averaging over range/velocity areas where no target is expected to fall. Clearly, the number of pixels constituting these areas across the available data files was kept sufficiently high as it sets the confidence interval for the estimation of the false alarm rate. Based on the global number of samples exploited, the margin of error is expected to be in the order of 2.5% for a 0.95 confidence coefficient.

The results when separately exploiting the four available frequency channels and after the MF integration schemes including various number of channels are reported in Table 2. Specifically, in the first columns of the table, the number of correct detections are reported for each data set and the bold font is used to highlight the best case identified for each different solution. The penultimate column of Table 2 lists the total number of correct detections summed up across all the available data sets so that it provides an overall analysis in term of target detection performance. Finally, the estimates of the false alarm rates are shown in the last column of the table in order to compare the capability to control the number of false alarms (the worst case margin of error has been evaluated to be smaller than 2.6% of the estimated value with a confidence coefficient of 0.95). First of all we observe that, consistently with Figure 6 and Figure 7, the estimated false alarm rates for the case of a MF operation are smaller and closer to the nominal *P_fa_* value. This especially applies to the decentralized integration strategy and to the centralized detection scheme operating with *N* = 3 or *N* = 4 DVB-T channels. This is likely to be due to the possibility to mitigate the disturbance background by averaging over the results obtained at different frequency channels. In contrast, when exploiting SF channels the actual false alarm rate can increase even by an order of magnitude.

Similarly, the detection capability is significantly enhanced when jointly exploiting multiple channels both with a centralized and with a decentralized detection strategy. Specifically, the centralized approach using all the available channels (CEN-MF-4) yields the best performance: it allows to increase the number of correct detections by 27% with respect to the best performing channel and it approximately doubles this number with respect to the worst performing channel. However, it is worth mentioning that the detection improvement of the CEN-MF-4 with respect to the CEN-MF-3 is extremely limited since one of the channels included in the MF integration provides a negligible contribution to the final performance. Therefore, in this case, the exploitation of just three of the available channels would be recommended in order to reduce the system complexity and the computational load.

Incidentally, we observe that the selection of good performing channels to be exploited by a multi-frequency detection scheme represents a key-point. In fact the inclusion of bad channels would be paid in term of increased system complexity without providing any improvement in the final performance. However an a priori selection could only be based on average performance (if a pre-screening of the available channels can be performed) or on typical parameters such as the transmitted power, the presence of co-channel and adjacent-channel interference, the transmission mode, etc. Some selection criteria along this line were defined in [23] for the FM radio case and could be successfully applied also to the DVB-T based passive radar case. Obviously, the a priori selection of good channels is not effective against the time varying behavior of each channel and this basically represents the main motivation for the proposed multi-frequency approaches.

## 6. Experimental Results for a Maritime Surveillance Application

The benefits resulting from the application of MF approaches for maritime surveillance are investigated with reference to an acquisition campaign that was carried out in a site located on a small Italian island.

The DVB-T PR receiver has been installed very close to the coast with the surveillance antennas pointed toward the open sea to allow the detection of the typical maritime traffic travelling during the day. The reference antenna was steered toward a DVB-T transmitter located about 170 km away. Only a single data set composed by 93 data files is considered in the following for this experimental campaign; each data file is about 1.2 s of duration with a temporal spacing of 12 s between two consecutive files. Therefore, a total acquisition time of 19 min is available. Four DVB-T channels have been simultaneously collected at the following carrier frequencies: 682 MHz, 634 MHz, 626 MHz and 546 MHz.

Although only the results for a single dataset are reported, we observe that many targets of opportunity were present in the surveyed area up to bistatic range of 210 kilometers so that the detection capability provided by different signal processing scheme can be reasonably compared.

**Figure 8:**
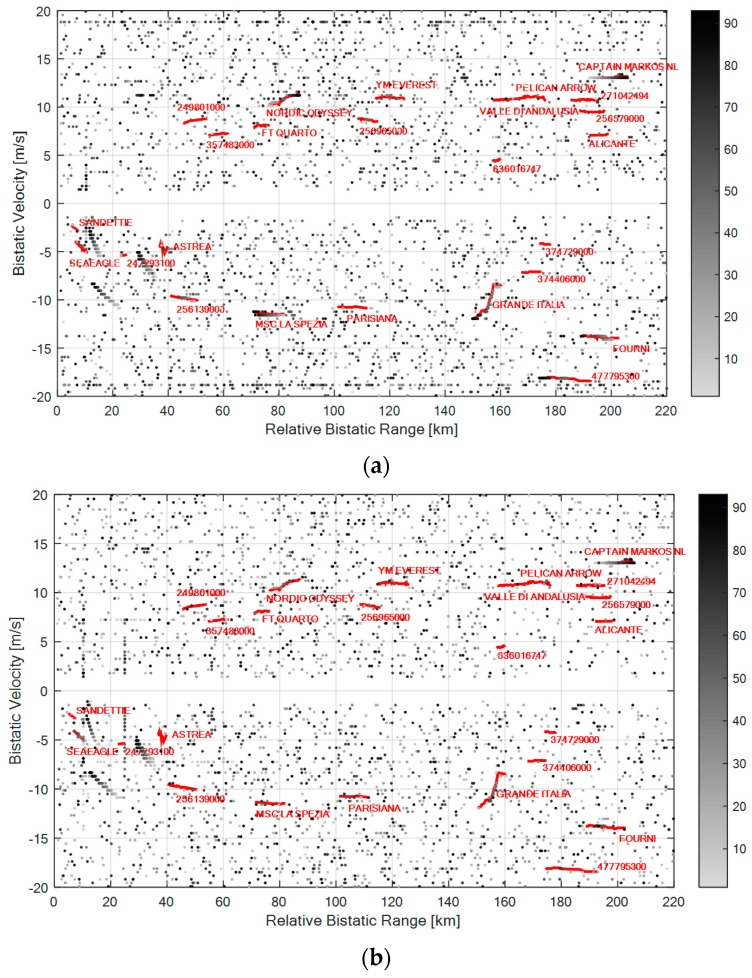
Detection results over the range-velocity plane using single DVB-T channels representing the best and the worst cases, respectively: (**a**) channel at 626 MHz; (**b**) channel at 546 MHz.

To this end Figure 8a,b show the detection results (grey dots) on the range-velocity plane for the best and the worst frequency channel, respectively. Again, a gray scale associated to the scan number has been used for the detections of the PR sensor. In this case, the ECA [24] is applied with 1000 taps and a CPI of 1.2 s is employed to evaluate the CAF maps over an area of 220 km × 20 m/s. Then a CA-CFAR with $P_{fa}=10^{-4}$ have been adopted (where Q and the guard interval are the same that have been defined previously) and a 2 out 2 detection criterion across the two available surveillance antennas has been applied to reduce the number of false alarms (namely $P_{fa}=10^{-8}$). In each figure, all the plots whose velocity in module was less than 1 m/s have been discarded to obtain much clearer outputs. The red tracks in Figure 8a,b identify the maritime traffic as provided by the Automatic Identification System (AIS) registrations.

As is apparent, with both the considered single channels, the system is able to detect many targets also at very long bistatic ranges (see for example the vessel labeled “Fourni” at about 200 km or “Capitan Markos NL” at 210 km). In fact, the conceived sensor was shown to provide OTH capabilities in the considered maritime application [19]. However, when exploiting single DVB-T channels, the false alarm rate is quite high, the detection performance is discontinuous along the target tracks and, possibly, different target tracks sections are detected by using different frequency channels. This in a measure points out the limitations of a SF operation and, in turn, highlights the diversity of information conveyed by multiple channels. These considerations are confirmed in Table 3 where a quantitative comparison is reported in term of correct detections provided for each of the available targets of opportunity.

As for the aerial surveillance scenario, by applying the proposed MF detection schemes, the results can be significantly enhanced. This is clearly apparent from the last rows of Table 3 where we reported the number of correct detections provided by centralized and decentralized MF approaches. The centralized technique is employed with *N* = 2, 3, and 4 channels while the decentralized DEC-MF-2/4 scheme is used with a range window size of *W* = 1, 3, and 5. In addition, in the last column is also reported the equivalent range resolution value (δr) obtained by degrading the range resolution (Δr) by a factor of *W*, namely δr = *W*·Δr.

We observe that, among the centralized configurations, the CEN-MF-3 yields the best performance as one of the integrated channel does not provide a remarkable contribution. It is worth noticing that the improvement with respect to the best performing single channel slightly reduces compared to the experimental tests reported in Section 4. This might be obviously due to a number of factors among which the particular bistatic geometry, the exploited transmitter of opportunity, the available DVB-T channels, etc. However, based on a detailed analysis of single target tracks, we verified that a limiting factor in this particular scenario is related to possible range misalignments of the target echoes received at multiple channels as discussed in Section 3.1.2.

**Figure 9:**
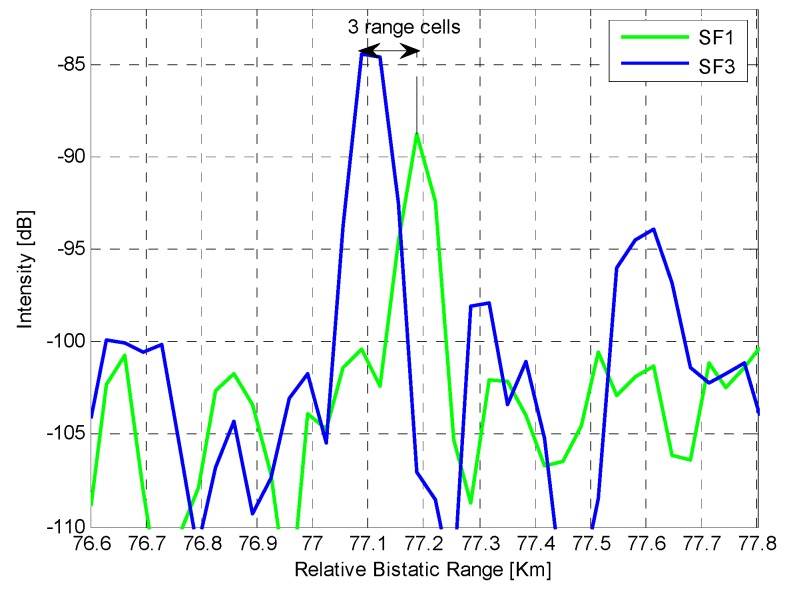
Range response at scan 45 of the target labeled “MSC La Spezia” when observed at two different DVB-T channels (enlarged view around the target bistatic range of the sections of the CAF extracted at the target velocity).

An example of this effect is shown in Figure 9 where we report sections of the CAF maps obtained, for a given scan, at two different DVB-T channels. Specifically, the sections extracted at constant velocity of −11.59 m/s are shown around the range location of the vessel labeled “MSC La Spezia”.

As is apparent, target range responses after matched filtering are centered around different bistatic ranges with a peak displacement of about 100 m. Notice that the considered target is a cargo ship so that its size is remarkable (365.82 m long × 51 m wide). However we point out that a similar effect was noticed also for other smaller targets when employing the same couple of channels which possibly reveals that these channels are emitted by spatially separated transmitting antennas. Therefore it is likely that a combination of the effects discussed in Section 3.1.2 occurred in this test campaign.

Obviously when such misalignment is present, the performance of the MF techniques rapidly degrades, especially when a decentralized detection scheme is applied as it operates after a binary decision separately performed on each channel. In contrast, the centralized MF approach is able to mitigate this effect since it benefits from the integration at intensity level so that even the tails of the target response could contribute to the enhancement of the detection capability. Nevertheless, in the final integrated map, the target could be smeared in the range dimension thus yielding a reduced multiple detections and/or a reduced accuracy.

**Figure 10:**
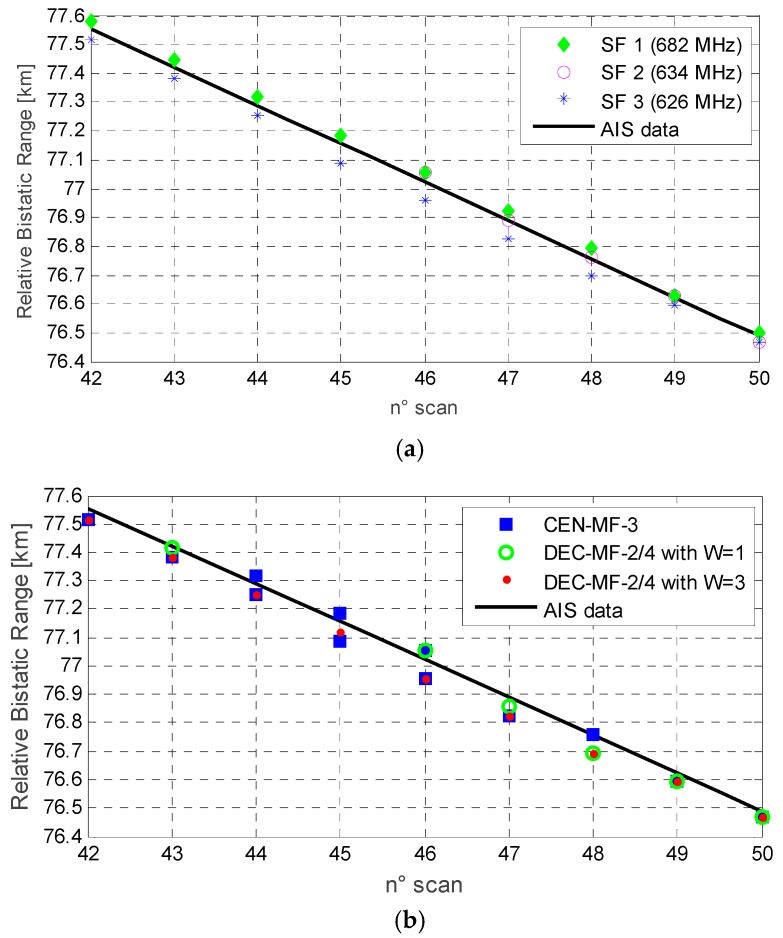
Detection results at few consecutive scans against the target labeled “MSC La Spezia” when (**a**) using SF channels; (**b**) when exploiting MF integration strategies.

Some examples along this line are reported in Figure 10 for the same target considered in Figure 9. In this case the raw detection results are shown as a function of the scan number by reporting the range coordinates of the obtained plots. Particularly, Figure 10a refers to the separate exploitation of SF channels and clearly shows the range misalignment of the resulting plots across consecutive observations. Consequently, Figure 10b reports analogous results after the application of different MF strategies. This figure highlights the detection loss (i.e., scan 42, 44 and 45) experienced by the decentralized approach when a unitary window is adopted in the range dimension. In contrast, the centralized approach is shown to be less sensitive to the observed misalignment that, in this specific case, is contained within a few range cells. Nevertheless, we observe that the scan 44, 45 and 46 yield two detections. Finally, the decentralized approach applied against a range window of three consecutive cells (i.e., *W* = 3), is even able to provide additional correct detections with respect to the best performing single channel.

According to the analysis above, the exploitation of a decentralized MF scheme operating over widened range windows allows one to partially recover the detection loss for targets affected by the discussed limitations. This is clearly apparent also from Table 3, where we observe that the DEC-MF-2/4 with *W* = 1 is not able to improve the performance with respect to the best performing single channel. In contrast, by using a window size *W* = 3, we obtain a number of correct detections slightly higher than the best channel and almost tripled with respect to the worst performing channel. Finally we notice that further increasing the window size W yields a corresponding degradation of the final performance due to the increase in the detection threshold needed to control the false alarm rate.

**Figure 11:**
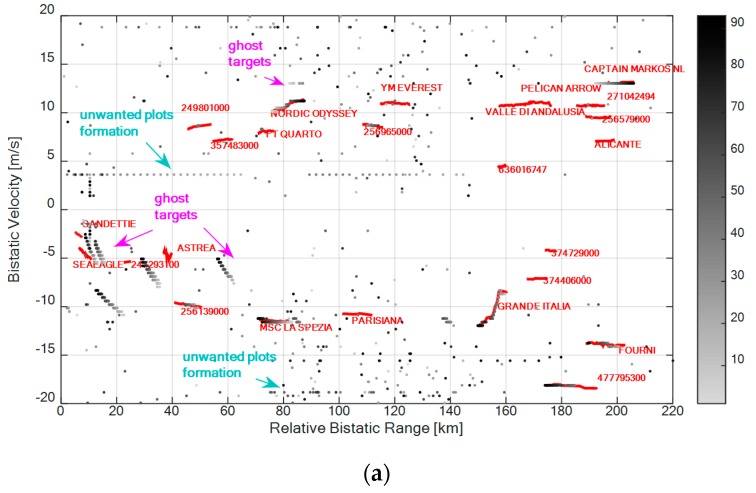

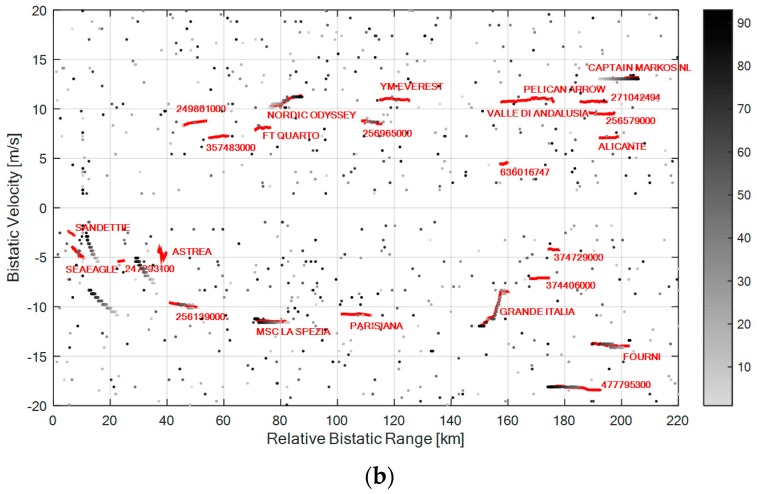
Detection results over the range-velocity plane for the best MF configurations: (**a**) CEN-MF-3; (**b**) DEC-MF-2/4 with *W* = 3.

To complete the picture, Figure 11 reports the detection results of the best centralized and decentralized MF approaches for the same case of Figure 8. Specifically the CEN-MF-3 and the DEC-MF-2/4 with *W* = 3 are considered in Figure 11a,b, respectively.

First of all we observe that a remarkable improvement is obtained with both the considered MF approach in term of false alarms control with respect to the results in Figure 8. We recall that, in this case, the detection threshold was set in order to obtain a nominal *P_fa_* = 10^−8^ after the 2-out-of 2 criterion on the available surveillance channels. However, the actual false alarm rate obtained in Figure 8 appears to be much higher.

The CEN-MF-3 approach guarantees a higher continuity in target detection performance since longer and much denser plot sequences are obtained against the targets of opportunity.

However, the DEC-MF-2/4 only yields a slight loss that does not prevent the possibility to track all the vessels navigating in the considered sea area.

Moreover the decentralized approach (see Figure 11b) provides the additional capability of suppressing the ghost target tracks and the unwanted plots formations that are present in Figure 11a when operating with the centralized approach. Such sequences of plots are usually yield by unremoved sidelobes of the signal AF in the presence of strong target returns. In addition they can arise from a frequency reuse by different transmitters in neighboring areas, there including the emitters of a single frequency network (SFN) transmission mode.

When multiple frequency channels are adopted, the undesired structures might appear at different locations of the resulting CAF maps. This is typically the case if the DVB-T channels employed exploit a different transmission mode and/or different modulation parameters [9]. Moreover it is unlikely that different channels are affected by the same interfering transmitter. Incidentally we observe that these aspects can be fruitfully exploited when selecting the DVB-T channels to be simultaneously employed in order to enhance the diversity also in term of AF characteristics and interfering scenario severity.

Apparently, when integrating the results at multiple frequency channels, provided that a certain degree of diversity can be guaranteed, possible unwanted outcomes will be filtered out regardless of the exploited MF approach. Obviously, a decentralized scheme could be much more effective even operating with few channels thanks to the two stages thresholding approach that does not retain the dynamic range of the single channels. In contrast, for a similar result to be obtained with a centralized scheme, the number of channels to be integrated should properly increase and this could represent a limitation in practical cases.

## 7. Conclusions

Appropriate MF techniques for target detection have been proposed in this paper for the case of a DVB-T-based PR sensor. Aiming at jointly exploiting the signals emitted by the same DVB-T transmitter on multiple carrier frequencies, the approaches presented in [23] have been properly modified and extended to be effective in the newly considered application.

Different non-coherent integration schemes have been considered, namely the centralized MF detection scheme and the decentralized MF detection scheme L/N. A detailed discussion of the required modifications that make them suitable for the exploitation of DVB-T broadcast transmissions has been presented. Specifically, the first modification concerned the selection of the integration time across the different frequency channels in order to obtain CAF maps directly comparable in the velocity dimension (namely, with the same velocity spacing). Furthermore, for the decentralized integration approach, a range resolution degradation has been proposed to counteract a possible range displacement of the target echoes received at different DVB-T channels. A preliminary performance comparison has been first carried on by means of simulations for specific case studies. Finally, the proposed MF integration schemes have been extensively compared against experimental data pertaining to aerial and maritime surveillance applications.

The obtained results clearly showed that the MF approaches yield a significant enhancement of the target detection capability of the PR sensor with respect to the SF operation. This improvement is not only related to the expected enhancement of the target echo resulting from the non-coherent integration. In fact, all the proposed MF integration schemes have been shown to also guarantee a better control of the false alarm rate thanks to their capability to average the characteristics of the channels simultaneously employed.

Among the proposed approaches, the centralized integration (operating with three or four DVB-T channels) typically yields the best performance in terms of target detection; in fact it always allows one to significantly increase the number of correct detections with respect to the best performing channel. Comparatively, the decentralized 2/4 approach yields only a slight loss in terms of target detection performance but provides the additional capability to mitigate the problems arising from the possible presence of ghost targets or unwanted plot formations (that are instead present in the centralized case). Therefore, based on the reported results, the decentralized integration scheme can be regarded as a practical solution in the considered applications if the target detection capability can be partly traded for a higher robustness to such problems.

## Figures and Tables

**Table 1:** Details of the datasets collected during the performed test campaign.

Dataset #	1	2	3	4	5	6	7
N° of data files	178	142	305	409	329	320	327
Total Time duration [min]	12	10	26	27	22	21	22

**Table 2:** Synthesis of the detection results obtained against all the available Data Sets for an aerial surveillance scenario.

	Data Set #1	Data Set #2	Data Set #3	Data Set #4	Data Set #5	Data Set #6	Data Set #7	Total Number of Correct Detections	Estimated False Alarm Rate
**SF 1** (586 MHz)	84	30	172	136	332	47	260	1061	1.06·10^−5^
**SF 2** (714 MHz)	**90**	**40**	**179**	**139**	**352**	**54**	**274**	**1128**	4.59·10^−6^
**SF 3** (762 MHz)	66	31	128	111	275	38	203	852	6.52·10^−6^
**SF 4** (634 MHz)	48	21	98	100	219	36	180	702	5.63·10^−6^
**DEC-MF-2/4**	**111**	**41**	**200**	**163**	**402**	**59**	**332**	**1308**	3.50·10^−6^
**CEN-MF-2** (SF 1-2)	111	42	215	173	425	63	335	1364	5.29·10^−6^
**CEN-MF-3** (SF 1-3)	**121**	43	**225**	**184**	434	67	356	1430	3.68·10^−6^
**CEN-MF-4**	120	**44**	224	178	**437**	**68**	**368**	**1439**	3.93·10^−6^

**Table 3:** Detection results with the single DVB-T channels and different MF approaches in the case of a maritime surveillance scenario.

	Target of Opportunity											Equivalent Range Resolution
	249801000	256139000	FT Quarto	Nordic Odyssey	256965000	Captain Markos NL	Fourni	477795300	Grande Italia	MSC La Spezia	Total Number of Correct Detections	
**SF 1** (682 MHz)	0	14	1	36	**11**	34	18	2	8	58	182	33 m
**SF 2** (634 MHz)	**3**	8	0	**70**	2	79	**37**	**42**	48	54	343	33 m
**SF 3** (626 MHz)	1	**22**	**5**	58	5	**89**	33	36	**55**	**56**	**360**	33 m
**SF 4** (546 MHz)	0	0	0	9	2	75	16	4	25	5	136	33 m
**DEC-MF-2/4, W = 1**	0	**19**	1	70	5	87	35	30	45	52	344	33 m
**DEC-MF-2/4, W = 3**	**2**	**19**	**2**	**72**	**10**	**88**	**42**	**44**	**53**	**63**	**395**	99 m
**DEC-MF-2/4, W = 5**	1	18	1	**72**	7	**88**	40	41	50	**63**	381	165 m
**CEN-MF-2** (SF 2–3)	3	26	**5**	**75**	8	**92**	39	43	**63**	70	424	33 m
**CEN-MF-3** (SF 1–3)	**5**	**28**	**5**	74	**9**	**92**	39	**44**	61	**81**	**438**	33 m
**CEN-MF-4**	0	25	**5**	72	6	90	**40**	**44**	57	74	413	33 m
